# Factors associated with loneliness among Chilean family carers of people living with dementia

**DOI:** 10.1002/alz.088528

**Published:** 2025-01-09

**Authors:** Claudia Miranda‐Castillo, Thamara Tapia‐Muñoz

**Affiliations:** ^1^ Universidad Andres Bello, Santiago Chile; ^2^ Millenium Institute for Research in Depression and Personality, Santiago Chile; ^3^ Millennium Institute for Care Research, Santiago Chile; ^4^ Millennium Nucleus on Sociomedicine, Santiago Chile; ^5^ University College London, London United Kingdom

## Abstract

**Background:**

The risk of developing loneliness in informal carers of people with dementia is high. Individual and cultural factors might play a differentiated role in the explanation of loneliness. The aim of this study was to describe loneliness levels in a group of family carers in Chile and its associated factors.

**Method:**

A sample of 283 Chilean carers was surveyed about loneliness (measured with the 3‐item of the revised UCLA scale) carer’s personality, and sociodemographic and health factors. Sociodemographic and clinical data from the person living with dementia was also reported by carers. Multiple linear regression models using bootstrap errors (1000 iterations) were carried out to determine predictors of loneliness.

**Results:**

Carers were, on average, 59 years old, mostly women (79%), married or in a partnership (59%), with children (78%), and caring for a parent (54%) with Alzheimer’s Disease (74%). The average level of loneliness was 4.33 (range: 3‐9), and 12% of carers reported problematic loneliness (³6). Forty‐two percent of loneliness variance was explained by emotion‐focused coping, age, extraversion, neuroticism, gain, social support, and depressive and anxiety symptoms. In a final model, gain (Coef. ‐0.050; 95% CI: ‐0.085 to ‐0.015) and emotion‐focused coping were negatively associated with loneliness (Coef. ‐0.028; 95% CI: ‐0.050 to ‐0.006), whilst depressive symptoms were positive associated with loneliness (Coef. 0.089; 95% CI: 0.050 – 0.129).

**Conclusions:**

Research usually focuses on negative aspects of caregiving. However, the perception of gain and emotion‐focused coping mechanisms used by carers can be protective factors against loneliness. Non‐pharmacological interventions might benefit from the development of those factors.

